# Development of Test Programs for the Biorelevant Characterization of Esophageal-Applied Dosage Forms

**DOI:** 10.3390/polym15163430

**Published:** 2023-08-17

**Authors:** Friederike Brokmann, Franziska Feindt, Werner Weitschies, Christoph Rosenbaum

**Affiliations:** Department of Biopharmaceutics and Pharmaceutical Technology, Institute of Pharmacy, University of Greifswald, Felix-Hausdorff-Str. 3, 17489 Greifswald, Germany

**Keywords:** esophagus, local drug targeting, biorelevant in vitro model, mucoadhesive polymer, films, peristalsis, saliva flow, esophageal transport

## Abstract

In the local treatment of the esophageal mucosa, the retention time of the different dosage forms, such as tablets, films or liquids, is of high relevance for the effective treatment of diseases. Unfortunately, there are only few in vitro models describing the esophageal route of administration. To predict the behaviour of an esophageal-applied dosage form, it is necessary to simulate the site of application in a biorelevant way. The aim of this work was to develop two test setups for an esophageal peristalsis model which was described in a previous study. Different parameters such as flow rate, peristalsis, angle of inclination or mucous membrane were varied or introduced into the model. A stimulated and unstimulated modus were developed and tested with two different dosage forms. The time until the dosage form was cleared from the in vitro model was shorter with the stimulated than with the unstimulated modus. Also, esophageal-applied films had a prolonged transit time compared to a viscous syrup. The modification of the simulated esophageal surface made it possible to estimate the retention time of the dosage forms. It could be demonstrated that the residence time of a dosage form depends on different parameters affecting each other.

## 1. Introduction

Diseases of the esophagus, such as eosinophilic esophagitis, have attracted increasing interest from medical researchers in recent years. Since the first description of eosinophilic esophagitis by Landres et al. in the 1970s, the prevalence in the population has risen sharply [1,2]. EoE is a chronic inflammatory disease of the esophageal mucosa caused by a variety of factors including food or airborne antigens, various environmental factors, or genetic mutations. Diagnostic criteria include the following:

1. Symptoms of esophageal dysfunction such as dysphagia, food impaction, chest pain or heartburn [3];
2. Endoscopic abnormalities such as fixed rings, longitudinal furrows, strictures, whitish exudates or crevices [4];
3. ≥15 eos/hpf in the esophageal mucosa [4];
4. Assessment of diseases in the esophagus that may cause EoE or have potential adverse effects [4].

The esophagus is the connecting organ between the oral cavity and the stomach and is approximately 25 cm long [5]. The collapsed muscular tube consists of both longitudinal and transverse striated musculature to which the submucosa is attached [5,6]. It contains blood vessels and small and large salivary glands and is innervated by the enteric nervous system. These salivary glands provide a constantly moist environment in the esophagus and are also able to neutralize reflux through the secretion of bicarbonate [7]. The submucosa is followed by the esophageal mucosa, a stratified squamous epithelium [5]. However, most of the saliva is not produced by the small glands in the submucosal layer, but by the three large salivary glands in the oral cavities, the parotid, submandibular and sublingual glands [8]. The amount and composition of saliva is highly variable, depending on the time of day, stimulation, posture, medications taken, or illnesses experienced [5,9,10]. Salivary flow varies from close to 0 mL/min, e.g., during sleep, with up to more than 6 mL/min when speaking, chewing or stimulating taste cells [5,10,11]. The esophagus is a slightly moist place anyway, but unlike other organs of the GIT, such as the small intestine with its water pockets, it is relatively dry, especially if the salivary glands in the oral cavity are not stimulated.

Esophageal peristalsis, the movement of food particles or fluids from the mouth to the stomach, is also very variable [5,12]. Primary peristalsis, which is triggered by conscious swallowing, is followed by secondary peristalsis, also known as “cleansing peristalsis” [13]. The frequency of peristalsis events varies according to the time of day and can occur between 100 and 600 times per day [5]. A peristaltic wave with a maximum pressure of up to 130 mmHg moves at a speed of 2–6 cm² from the upper to the lower esophageal sphincter [5,14]. During sleep, the number of peristaltic events decreases to less than 10% of daytime activity [5,15].

The treatment of patients diagnosed with EoE can be based on three main approaches: the elimination of the triggering allergens from the diet by means of a special diet, PPI therapy, and the use of topical glucocorticoids [16].

The residence time of the drug at the site of action is crucial for local therapy of the esophagus [17]. This problem must be considered when applying a drug because of the anatomy of the esophagus. Drugs, food components or liquids pass through the esophagus in a matter of seconds [18]. This is problematic for a long contact time of an active substance [19,20]. There are several concepts that lead to prolonged transit time in the esophagus [21], such as various syrups or viscous suspensions and solutions [22] or orodispersible tablets [23]. The placement of mucoadhesive films in the esophagus, as made possible by the novel EsoCap-technology, is another innovative concept for local and targeted therapy [24].

To assess how such dosage forms will behave in vivo, it is necessary to carry out preliminary studies on in vitro models. It is important to reproduce the physiological conditions at the site of application as accurately as possible. Especially in the early phases of drug development, biorelevant in vitro test systems can provide valuable information on the performance of dosage forms. Many well-established in vitro test systems now provide a very efficient and cost-effective means of mapping the specific properties of each site of application.

Using a new esophageal model that simulates esophageal peristalsis, different body positions and salivary flow, the residence time of mucoadhesive films was previously studied at different flow rates, model tilt angles, peristaltic wave velocities and number of peristaltic events [25]. These initial experiments showed that model tilt, number of peristaltic events and flow rate affected the retention time of mucoadhesive films. The aim of this work was to create biorelevant setups, representing a stimulated mode (high salivary flow, many peristalsis events, upright position) and an unstimulated mode (low flow, few peristalsis events, supine position). In particular, the aim was to simulate the possible extreme conditions of the esophagus during the course of the day in order to characterize and compare dosage forms aimed for esophageal targeting according to these scenarios. In addition, the permanent slightly moist environment in the esophagus should be simulated in an appropriate way to also increase the biorelevance of the model.

## 2. Materials and Methods

### 2.1. Materials

Polyvinyl alcohol 18-88 (PVA) was kindly provided by Merck (Darmstadt, Germany). Glycerol as plasticiser was purchased from Caelo (Hilden, Germany). Demineralised water was used as solvent. Fluorescein sodium as model substance was purchased from Sigma-Aldrich Chemie (Darmstadt, Germany). Potassium dihydrogen orthophosphate was obtained from neoFroxx GmbH (Einhausen, Germany), sodium hydroxide was purchased from AppliChem GmbH (Darmstadt, Germany) and honey was obtained from Langnese Honig GmbH & Co. KG (Bargteheide, Germany). PVA strings were purchased from M&R Angelgeräte GmbH (Karlsruhe, Germany).

### 2.2. Methods

#### 2.2.1. Film Preparation

The films were manufactured using the solvent cast evaporation method. The polymer films, 25.0 cm long and 0.4 cm wide, were prepared using 18.0 g polyvinyl alcohol (PVA type 18-88), 2.0 g glycerol (anhydrous) and 80.0 g purified water as the solvent. As a model drug, 0.04 g fluorescein sodium (FS) was added. All components were mixed with a magnetic stirrer at 500 rpm in a laboratory glass bottle and then heated in a water bath at 80 °C for two hours with continuous stirring at 100 rpm. After adding FS, the mixture was stirred continuously at 80 °C for a further 60 min. For reasons of stability, the following steps were carried out in the absence of light. The mixture was stirred at 50 rpm overnight and cooled down to produce a bubble-free mixture. If the mixture was not completely free of air bubbles, it was centrifuged at 4400 rpm for 15 min at 20 °C to obtain a homogeneous and bubble-free mixture.

The prepared polymer solution was then applied to a motorised film-casting apparatus (mtv messtechnik CX4, Erftstadt (Gymnich), Germany). It was applied homogeneously to a polyethylene coated liner using a square applicator (gap height: 1000 µm). The film was dried at room temperature over night before testing. The films were then cut into elongated pieces of 25.0 cm × 0.4 cm and stored in amber glass bottles at room temperature in the absence of light.

The thickness of the films was measured using a mechanical dial gauge (0.01 mm capacity, Kaefer Messuhrenfabrik GmbH & Co. KG, Villingen-Schwenningen, Germany). In addition, ten samples (size 10 cm × 0.4 cm) were randomly selected, weighed and dissolved in 100.0 mL phosphate buffer pH 7.4 USP. The samples were spectrophotometrically analysed using a fibre-optic-based system (Cary^®^60, Agilent Technologies, Santa Clara, CA, USA) equipped with 5 mm slits at 491 nm against a baseline correction at 600 nm. The maxima of the absorbance and the linearity of the absorbance range were determined. The analytical method was validated with respect to linearity, precision, accuracy and selectivity.

#### 2.2.2. Honey Syrup

The honey syrup was used referring to Hefner et al. who investigated the clearance of highly viscous slurries [22]. The publication by Hefner et al. does not provide precise information on the viscosity of the syrups used, which may vary due to the fact that they are natural products. Since Dellon et al. investigated the differences between different viscous carriers in their publication, it was decided to use a syrup with a viscosity as high as possible, which would allow homogeneous incorporation of the active ingredient and thus result in as little dilution of the honey as possible [17]. A syrup was therefore prepared from 90% linden blossom honey (Lidl Stiftung & Co. KG, Neckarsulm, Germany) and 10% aqueous fluorescein solution, resulting in a drug load of 2 mg/mL in the honey syrup, which allowed easy detection by UV/Vis analysis. The viscosity of the drug-loaded syrup was 52.3 cP and was determined using a Brookfield RVDV-II+CP rotational viscometer (Brookfield Engineering Middleboro, MA, USA) at 20 °C.

#### 2.2.3. EsoPeriDiss Model

**Figure 1:**
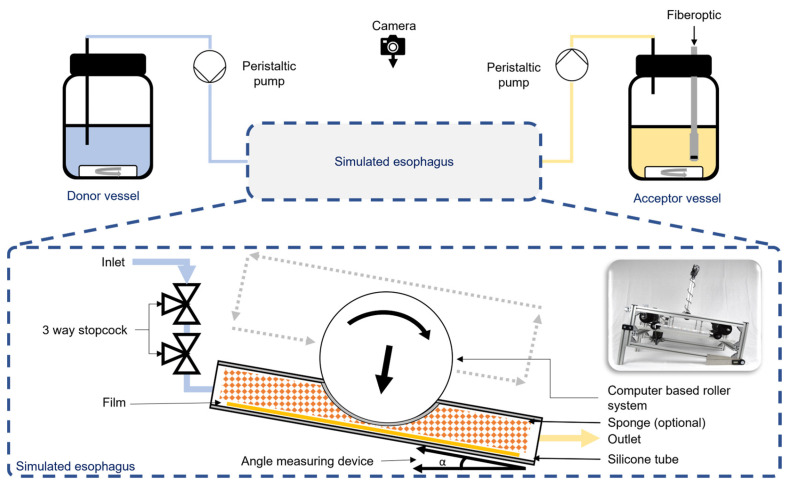
Schematic experimental setup of the Esophageal Peristalsis Dissolution-Tester (EsoPeriDiss).

The Esophageal Peristalsis Dissolution-Tester, or EsoPeriDiss (EPD) for short, is a biorelevant model that can be used to simulate the esophagus to characterise the retention time of an oral dosage form targeting the mucosa of the esophagus (Figure 1). It is possible to simulate the number of peristaltic events, the amount of saliva flowing into the eso-phagus and the position of the simulated subject.

The esophagus itself is simulated by a silicone tube with an internal diameter of 5 mm and a wall thickness of 1 mm. Rollers attached to a belt can be moved over the silicone tubes by an electric and computer-controlled motor (Nema 24, Nanotec, Feldkirchen, Germany). This enables a compression of the flexible tubes to simulate biorelevant peristalsis of the esophagus. The computer-based roller system offers the opportunity to create different states of the esophagus. Several parameters, in particular the esophageal peristalsis, are highly variable throughout the day and depend on the frequency a person is swallowing.

To be able to represent the highly variable saliva flow rates in humans over the course of the day in a biorelevant way, a peristaltic pump (ISM932 D, IKA-Werke GmbH & CO., KG, Staufen, Germany) was used to pump the saliva simulation medium from a temperature-controlled storage vessel into the release model itself. Flow rates of 0.5 mL/min to 30.0 mL/min could be simulated.

A second peristaltic pump (ISM833 C IKA-Werke GmbH & CO. KG, Staufen, Germany) was installed at the outlet of the in vitro model. The flow rate of the peristaltic pump was set to 30 mL/min. This pump was used in addition to enable the transit of the dissolution medium through the model and to avoid an unphysiological accumulation of the dissolution medium in the EPD.

In order to avoid negative pressure in the silicone tubing and the possibility of different travel times through the test system, two three-way valves were installed in front of each inlet of the model. The 3-way valve closer to the first pump was used exclusively to equalize the pressure in the system and was always open to allow air to enter the system if necessary. The second 3-way tap was used to apply very small amounts of liquid to the EPD. These liquids could be solutions of the active ingredient to determine the LAG time for different dosage forms, or different dosage forms themselves, which will be described later in detail.

The dissolution medium containing the dissolved drug was pumped via commercially available Heidelberger extensions, one meter in length, into an acceptor vessel where the drug concentration could be measured using an installed fibre-optic UV/Vis system. The acceptor vessel was filled with a defined amount of dissolution medium to determine the amount of drug released. Based on the test setup, the quantity of retained dose in the silicone tubes was calculated backwards from the measured concentration of active ingredient in the acceptor vessel. In addition, a UV light system combined with a camera (Logitech C270 HD, Logitech, Lausanne, Switzerland) enabled the visualization of the retention of the dosage form due to the fluorescent properties of the model drug FS.

Although the esophagus is a comparatively dry site compared to the small intestine, it is always slightly moist. Small mucosal glands located in the esophageal mucosa secrete small amounts of saliva and mucus, even during the night when the large salivary glands in the oral cavity are mostly inactive. To represent this moist environment, it was possible to insert a suitable 30 cm long open-pored polyurethane sponge (flexolan e.K. Versandservice, Diedorf, Germany) into the model.

In addition, the model could be operated both in an upright and horizontal position to be able to represent the essential body postures of humans throughout the day. All experiments could be run simultaneously as triplicates.

#### 2.2.4. Development of a Stimulated and Unstimulated Test Program

Two different test scenarios, which should describe physiological parameters in the esophagus throughout the day, were designed. With these test setups, it should be possible to predict the clearance of drug forms from the esophagus in a biorelevant way. In this context, clearance means the time needed for the applied drug formulation to be dissolved and removed from the site of action. One scenario simulates physiological parameters in the esophagus during daytime with a high flow rate and high peristaltic activity. That should have a negative impact on the retention of drugs according to previous studies. The second scenario simulates a low flow rate und low peristaltic activity to predict the retention of drug forms during night. These unstimulated conditions, on the other hand, should have a positive effect on the retention according to previous findings.

The construction of the peristalsis was based on literature data on the velocity and frequency of peristaltic events in the esophagus. During the day, humans swallow up to 600 times and every swallow is followed by a peristaltic event [5]. The movement of the stepper motor was programmed to move the fixed roller over the silicone tubes 180 times per hour. The velocity was set to 3.7 cm/s based on previous studies with the used in vitro model and on literature data that described the speed of a peristaltic wave to be between 2–6 cm/s [5,25]. The flow rate was assessed as 6.0 mL/min according to the literature to simulate an extreme secretion of saliva [26]. The model was placed at an angle of 90° to simulate an upright body position. For the unstimulated scenario, the frequency of peristalsis events was reduced to 6 per hour because the rate of swallowing during sleep is reduced to less than 10% of the daytime swallowing rate [5]. This was achieved by programming the motor controller so that the speed of the simulated peristaltic events was constant in all cases. The flow rate was reduced to 0.5 mL/min to simulate a basal secretion during the sleep whereby this flow rate represents the upper limit of this value according to literature data. The model was placed at an angle of 0° to simulate a supine body position. All parameters for the stimulated and unstimulated setups are shown in Table 1.

#### 2.2.5. Experimental Procedure

At the beginning of each trial, the peristaltic pumps used were calibrated according to the manufacturer’s instructions. Before the mucoadhesive films were tested, an insoluble polyester thread using a placebo PVA mixture was fixed at one end of the films to pull them into the silicone tubes. For the test scenarios with the open-pored sponges they were also fixed to the polyester threads and pulled into the silicone tubes together with the films.

The tubes were inserted into the dissolution model so that the films were centred under the roller system. The pumps were then connected to the system via the Heidelberg extensions as already described and 240 mL of release medium was introduced into the acceptor vessels. The following steps differed as follows when examining the film and syrup dosage forms: for the films, a syringe with 10 mL of release medium was connected to the first 3-way stopcock that was not intended for pressure equalization, and the measurement was started at the moment of applicating the dissolution medium into the system.

For the syrupy dosage forms, a syringe with 10 mL dissolution medium was also connected to the first 3-way stopcock and applied in bulk to moisten the entire system, especially the open-pored sponge when it was used. Afterwards, the syringe was immediately exchanged for a syringe with the spiked syrup. The measurement to characterise the syrup began with the mass application of the syrup to simulate swallowing the dosage form.

The retention of the dosage forms and LAG time were analysed for the stimulated scenario over 30 min and in unstimulated modus over 90 min. The concentration of the model drug was measured in the acceptor vessel every minute using the UV/Vis fibre-optic method described earlier at 491 nm versus 600 nm baseline.

#### 2.2.6. Latency

The construction of the in vitro model is accompanied with a delayed detection of the model drug in the acceptor vessel. When the measurement is started with application of the syrup dosage form or defined amount of dissolution medium as described above, the detection of the first fraction of the model drug is delayed by the time the dissolution medium needs to dissolve the active substance and pass the in vitro model. The so-called latency was determined for each scenario in the stimulated and unstimulated modus with and without a sponge. Experiments were carried out according to the tests with the syrup-like dosage forms. Instead of applying the syrup to the in vitro model, 100 µL of an FS-containing stock solution were administered via the 3-way valves in each of the three silicone tubes and the measurement was started. This marked the time t = 0. The LAG time then resulted from the transit time of the small amounts of FS solution until detection in the acceptor vessel.

## 3. Results and Discussion

### 3.1. EsoPeriDiss

The EsoPeriDiss in vitro model described by Rosenbaum et al. offers the possibility to configure different parameters depending on each other [25]. The modular concept of the model enables to set up these parameters to represent the basic characteristics of the esophagus described in the literature [25]. For this purpose, it was possible to arrange the device parameters shown in Table 1 and to develop a stimulated and an unstimulated mode. Therefore, in particular the flow rate, the number of peristaltic events, the speed of the individual peristaltic waves and the body posture were considered in the development of the modes.

### 3.2. Flow Rate

The flow rate can be adjusted via the first peristaltic pump that delivers the dissolution medium from the donor vessel to the EPD model. The flow rates of 0.5 mL/min and 6.0 mL/min represent literature values, whereby in some cases, both lower and higher flow rates have been described in the unstimulated and stimulated esophagus, respectively [5,9,10]. The flow rate for the unstimulated mode was set at 0.5 mL/min to simulate a basal flow rate as occurs during sleep [5]. In principle, salivary flow from the large salivary glands in the oral cavity largely stops during the night. However, there are additional small salivary glands in the esophageal mucosa that permanently produce a small amount of highly viscous saliva/mucus during the night to moisten the mucosal surface [5].

Due to the power range of the peristaltic pump to be calibrated, flow rates significantly below 0.5 mL/min were also challenging to demonstrate. The saliva flow rate plays an important role in the retention of an esophageal-applied drug formulation [25]. A large volume of dissolution medium is able to dissolve a higher amount of the applied drug formulation as shown in the previously published data of Rosenbaum et al. They investigated the isolated influence of doubling the flow rate from 0.5 mL/min to 1.0 mL/min on the retention of films administered to the simulated esophagus [25]. Furthermore, a biorelevant in vitro model such as EsoPeriDiss is a useful tool to characterize different drug formulations, especially in early phases of development. In vitro models are very convenient for evaluating, characterizing, and comparing different dosage forms, because of their standardization, reproducibility, and low costs on operation. A low salivary flow rate of 0.5 mL/min is a good compromise for starting with characterization of new dosage forms. It is very unlikely that salivation will be reduced significantly or stop after applying a swallowed dosage form to esophagus, especially after the application of a flavoured syrup that could increase salivation. To increase the biorelevance of the in vitro model, the flow rate could be reduced even further over time. That would lead to a negative effect on liquid or semisolid dosage forms such as syrups or gels as these would be flushed out quickly because these do not have to be dissolved first. Drug formulations with a slow release of drug would show a prolonged retention due to the lower flow rate. A lower amount of dissolution medium can dissolve a smaller fraction of the applied dosage form and release of the drug. Also, the time needed for the solvent to pass the EPD is extended. For the stimulated mode, the flow rate was set at 6.0 mL/min, which corresponds to a very high salivary flow during eating or when acidic stimuli are applied to the tongue. Literature data suggest salivary flow rates even above 6.0 mL/min [5,10,11]. However, these are difficult to represent in the EPD due to the model design. The size of the acceptor vessel is a limiting factor, and the homogenization and analytical determination would be difficult to handle. This high flow rate was chosen to demonstrate the retention behaviour of a locally applied drug in the esophagus under extreme conditions, whereby such a high flow rate over a longer period can be considered unphysiological.

### 3.3. Speed/Peristalsis

The computer-controlled motor system allowed the simulation of many different scenarios whereby two characteristic scenarios were to be compiled. In combination with the simulated salivary flow rate, these scenarios were to simulate the physiological extremes during the day. The number of peristaltic events showed a clear influence on the release in the previously published data by Rosenbaum et al. [25]. In previous work, the speed was always varied in addition to the number, which led to an increase in the number of events. In the present work, deliberate emphasis was placed on a physiological and thus biorelevant simulation of the speed of the peristalsis, which was easy to achieve through computer-based motor control. Corresponding holding times of the roller system in the unloaded state of the model were determined to ensure that the rollers would not have been able to press down the simulated esophagus for a longer period at any time [5,6,27]. This could also have potentially led to a fluid build-up and thus to non-physiological conditions. The speed of the motor-driven roller system was 3.7 cm/s, based on the literature, suggesting an esophageal peristaltic wave speed of 2–6 cm/s [5,14].

Under physiological conditions, people swallow up to 600 times a day where 1/3 of all events occur while eating or drinking and less than 10% of swallows occur during sleep [5]. Based on 8 h of sleep for an adult, approximately one swallow and corresponding peristaltic event would occur every ten minutes. Based on this assessment, the frequency of the motorised roller system was set at six peristaltic events per hour for the unstimulated mode and was intended to represent the nocturnal events in particular. The modus was supplemented with the low salivary flow rate of 0.5 mL/min and a horizontal, i.e., lying, experimental setup.

During a day when a person is awake and not eating or drinking, we estimate that about 350 swallows and peristaltic events occur in the esophagus. This equates to one peristaltic event every 20 s. We therefore calculated 180 peristaltic events per hour for the stimulated mode, which was supplemented by a high flow rate and a vertical, upright test setup. The peristaltic waves were evenly distributed over time so that in the unstimulated mode, a pressure event occurred every 10 min. Possible physiological and in vivo variables, such as the occurrence of secondary peristaltic events due to a cleaning reflex, which could be caused by local application of the dosage forms in the esophagus, were not considered. The model does not have an assessment of the dosage form in the area of induction of secondary peristalsis events, although it is conceivable that with an expanded database, the number of simulated peristaltic waves due to a triggered cleaning reflex could be variably adapted to the dosage form to be characterised, especially in the unstimulated mode. Spontaneously occurring spasms or tertiary peristaltic events are also not included in the model setup, as the necessary data base is not yet sufficiently presented in the literature to establish these phenomena in an in vitro model.

### 3.4. Angle of Inclination

As mentioned above, in addition to the flow rate and the number of peristaltic events, the angle of inclination of the experimental setup was also varied to match the physiological conditions. For the stimulated mode, the angle of the model was set to 90° to simulate an upright body position. The supine position in the unstimulated mode is simulated with an angle of 0° to represent a sleeping position [9]. The effect of body position on esophageal transit has been described many times in the literature. In the upright position, the effect of gravity also influences the disruption of a film. Rosenbaum et al. investigated the effect of model positioning on film dissolution. They described a correlation between the positioning of the model and the retention time of the film in the in vitro model. Simulated upright body positions (90°) led to an increase in the retention time of a film, whereas a flat angle (0°, 10°) led to a decrease in the retention time of a film.

It should be noted that, in their initial investigations, Rosenbaum et al. deliberately varied individual parameters of the model in isolation to characterize the model. However, this very attractive possibility of studying individual parameters in isolation is only possible in vitro. In vivo, under physiological conditions, such phenomena cannot usually be observed; usually parameters such as salivation or body position affect each other. Therefore, it was important to present, if possible, two characteristic scenarios simulating two particularly contrasting esophageal conditions. For this reason, it is important to consider other parameters such as flow rate and number of peristaltic events when studying esophageal clearance. In the present study, the results show that a 90° angle combined with a high flow rate and a high number of peristaltic events leads to a faster clearance of a film from the EPD model, whereas a 0° angle, a low flow rate and a lower number of peristaltic events leads to a reduced clearance of a film.

### 3.5. Mucous Membrane

In addition, the introduction of thin, open-pored sponges was intended to allow permanent moistening of the dosage forms applied to the model. Furthermore, a structural element within the silicone tubes simulating the esophagus could be established. The slightly moist sponges in the EsoPeriDiss do not, of course, correspond to the physiological small mucous cells found in the esophageal mucosa [5]. It is difficult to simulate small salivary glands located in the esophageal mucosa; so, the salivation is only represented by the simulated oral cavity via the peristaltic pump. In particular, the viscous solution normally secreted by the mucous glands cannot be simulated with the phosphate buffer USP pH 7.4 used to date. Other possibilities for simulating the esophageal surface, such as the use of structured agarose gels, have been discussed and investigated in preliminary trials. However, the sponge-like structure showed great advantages, including the fact that this sponge-like structure was flexible and insensitive to pressure, and could be easily placed in the simulated esophagus with the thread used. In addition, the pressure-insensitive sponge structure was not washed out of the model, did not lead to accumulation of the drug, which could have resulted in incomplete release, and did not interfere with the analytical methods used.

### 3.6. Further Three-Way Stopcock/Syrup/Initial Media Delivery/LAG-Time

The addition of a supplementary three-way stopcock per simulated esophagus in the inlet area of the model was also intended to allow the application and characterization of liquid and semi-solid dosage forms by means of EsoPeriDiss. It was also possible to characterize the initial wetting of the films, which corresponds, for example, to the usual application technique of the EsoCap concept, without negatively affecting the venting or pressure equalization within the model. In addition, the initial wetting of the system, especially in conjunction with the use of the open-pore sponges at low flow rates, was intended to prevent a potentially extremely long LAG time that could have resulted from the wetting of the sponge structure. Despite the initial wetting, the measured LAG time was extended with the sponge inserts compared to the setups without the sponge structure. In addition, the water absorption capacity of the sponge led to a temporal retention of the released medium, resulting in a characteristic release profile, especially in the unstimulated mode, in which the regularly occurring peristaltic events were clearly visible.

The peristalsis model used, and the scenarios compiled represent a possible in vitro scenario that could also occur in vivo in the same or a similar way, although in vivo a mixture or changing scenarios must be expected. Further in vivo data, e.g., from telemetric capsule systems, would be of great interest in order to further adapt the model and possibly integrate data from patients with esophageal diseases into the model. The use of artificial saliva should also be investigated and the influence of different saliva viscosities over the course of the day cannot be represented with the current model.

### 3.7. In Vitro Test

**Figure 2:**
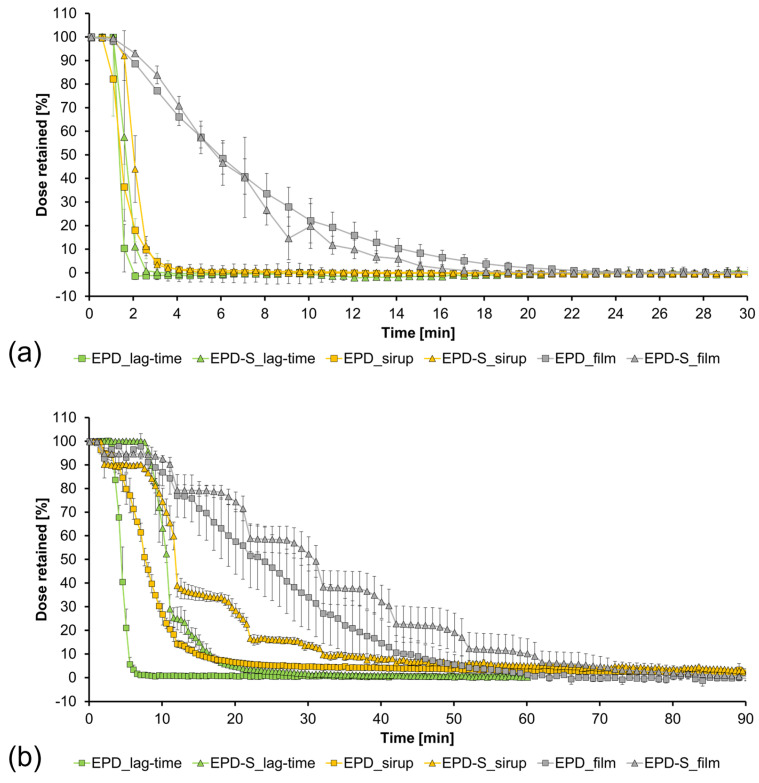
Lag-time and dissolution of a film and honey syrup with and without a sponge-like insert: (**a**) stimulated modus, (**b**) unstimulated modus (n = 3, +/− SD) EPD = EsoPeriDiss, EPD-S = Eso-PeriDiss with sponge.

The results of the in vitro tests are presented in Figure 2a,b as a reverse dissolution profile showing the time taken for the model drug FS to dissolve and pass through the in vitro model. For a better understanding of the time the model drug is present in the in vitro model, the LAG time was investigated.

For the stimulated scenario in Figure 2a, more than 90% of the FS solution is removed from the EPD after 2 min. The film (EPD_film) shows a prolonged and uniform dissolution with 90% of the FS cleared from the EPD after 15 min in the stimulated scenario. In comparison, Figure 2b shows the LAG-time and dissolution behaviour under unstimulated conditions. Both LAG-time and retention time of the film are prolonged. An amount of 90% of the FS solution has passed the EPD after 6 min and the retention time for the film has also been increased to 45 min for 90% to be removed from the EPD model. These results show that a combination of varying flow rate, number of peristaltic events and angle of the in vitro model resulted in different clearance times of a locally applied dosage form. Changing these parameters affects the retention time of an applied polymer film.

The honey syrup was inserted into the EPD via the first three-way stopcock. Both the peristaltic pumps and the roller system were started at the same time with the application of the honey syrup. For the test scenarios with the open pored sponge 10 mL of dissolution medium was added to the model before the syrup was applied to simulate a moist environment. In Figure 2a, the clearance of the syrup is as fast as the LAG-time. In both cases, 90% of the LAG-time solution and syrup were cleared from the EPD within three minutes. The increased viscosity of the syrup has no impact on the retention time in stimulated modus in the EPD with and without a sponge. Figure 2b shows the results of the dissolution of the film and honey syrup and LAG-time with the unstimulated modus in the EPD with and without a sponge. LAG-time and dissolution of the honey syrup is prolonged in the EPD with the sponge. After 32 min, 90% of the syrup is cleared from the EPD with a sponge. On the contrary, 90% of the solution for LAG-time is cleared from the EPD with a sponge after 18 min. These dissolution profiles also show plateaus at the same time points and a decrease in the retained dose at the end of the plateau. The plateaus describe a removal of the dosage from the in vitro model when a peristaltic event occurs. There is a difference in the shape of the plateaus between the film and the syrup due to different dissolution behaviours. The film must be moisturized to form a highly viscous gel which is then cleared from the in vitro model. The syrup is already a liquid viscous formulation. The viscosity has also an effect on the retention behaviour of the three different application forms. The higher the viscosity, the higher the retention time in the non-stimulated scenario.

The investigation of the clearance of different application forms is based on previous studies for the development of a treatment for EoE. Hefner et al. developed different viscous slurries to enhance the contact time of a drug to the esophageal mucosa [22]. The results from the in vitro model show that higher viscosities of such slurries lead to a prolonged clearance when compared to a solution. However, different parameters, e.g., flow rate, peristalsis, and positioning must be considered when the performance of a new application form is evaluated.

Based on the available in vivo data from Hefner et al. and the basic feasibility of using honey syrup as a reference for the evaluation of new dosage forms, a clear advantage of topically applied films over syrups could be established [22]. However, it should be noted that honey syrup is a natural product that may be subject to viscosity fluctuations due to natural conditions.

## 4. Conclusions

The esophagus is a complex site for drug delivery. There are several parameters that can influence the residence time of a locally delivered drug. Based on previous experiments, we developed two test scenarios simulating esophageal conditions during daytime when the patient is awake and during nighttime when the patient is asleep. These scenarios can be used for investigating clearance of various routes of administration for local esophageal treatment. By adjusting different parameters such as flow rate, peristalsis and the positioning of the model, two test scenarios could be created. In addition, the above-mentioned in vivo variability of peristalsis, flow rate, mucosal influence and possible irritation of the esophagus must be considered, and therefore the inclusion of new in vivo data from the esophagus would be highly relevant for further development. Despite all the limitations of in vitro biorelevant test systems, it is important to recognize that they are a major advantage, particularly in the early stages of formulation development, allowing very cost-effective initial assessments of the performance of new dosage forms at a very early stage. Furthermore, this leads to a better understanding of the processes at the site of administration and could therefore be used to predict the performance of different dosage forms in in vivo studies. The developed test programs allow the combination of different parameters to simulate complex conditions in the esophagus.

## Figures and Tables

**Table 1:** Overview of the parameters for the stimulated and unstimulated test scenarios for the EsoPeriDiss.

	Stimulated	Unstimulated
Flow rate	6.0 mL/min	0.5 mL/min
Speed of peristaltic wave	3.7 cm/s	3.7 cm/s
Number of peristatic events	180/h	6/h
Angle	90°	0°
Modification through open-pored sponge	Yes/No	Yes/No

## Data Availability

The data presented in this study are available on request from the corresponding author.
